# Activation of Rubber-Seed Shell Waste by Malic Acid as Potential CO_2_ Removal: Isotherm and Kinetics Studies

**DOI:** 10.3390/ma13214970

**Published:** 2020-11-04

**Authors:** Azry Borhan, Suzana Yusuf

**Affiliations:** HICoE, Centre for Biofuel and Biochemical Research, Institute of Self-Sustainable Building, Department of Chemical Engineering, Universiti Teknologi PETRONAS, Seri Iskandar 32610, Perak, Malaysia; drsuzana_yusuf@utp.edu.my

**Keywords:** rubber-seed shell, activated carbon, malic acid, CO_2_ adsorption, thermodynamics, kinetics modeling

## Abstract

Carbon dioxide (CO_2_) has been deemed a significant contributor to the climate crisis and has an impact on environmental systems. Adsorption is widely used among other technologies for carbon capture because of its many benefits. As a starting material for the production of activated carbon (AC) by chemical activation using malic acid due to its biodegradable and non-toxic properties, rubber seed shell (RSS) was used as agricultural waste from rubber farming. Sample A6, which was carbonized for 120 min at a temperature of 600 °C and impregnated at a ratio of 1:2, was identified to achieve the highest surface area of 938.61 m^2^/g with micropore diameter of 1.368 nm, respectively. Using the fixed volumetric approach measured at 25, 50, and 100 °C, the maximum CO_2_ adsorption capability reported is 59.73 cm^3^/g of adsorbent. Using the pseudo-first order of Lagergren, the pseudo-second order and the Elovich model, experimental data is modeled. It appears that, based on the correlation coefficient, the pseudo-first order model is aligned with the experimental findings. Furthermore, the activation energy of under 40 kJ/mol indicated a physical adsorption occurs, indicating that the RSS chemically activated with malic acid is a fascinating source of CO_2_ removal requirements.

## 1. Introduction

According to the Global Carbon Capture & Storage (CCS) Institute [1], the world’s leading CCS think tank, carbon dioxide (CO_2_) is a ubiquitous existence in the earth’s atmosphere with the characteristics of being colorless and odorless. The gas is a part of greenhouse gas (GHG) with a significant function in maintaining the global temperature by trapping the energy from the sun. However, the current emission in the 21st century has gained an international concern with the rise in the climate temperature due to an excessive release of CO_2_. The high demand for energy usage in industrialization and inhabitants in many countries have causes excessive and inefficient hydrocarbon use that in turn releasing immense concentrations of anthropogenic CO_2_ into the atmosphere, consequently, disrupts the planet‘s carbon balance. Practically every division of the worldwide economy, from assembling to agrobusiness to transportation to energy generation, contributes GHG to the atmosphere. It has been recorded that global CO_2_ concentrations have been increasing from 280 ppm during pre-industrial revolution to nearly 415 ppm nowadays [2]. This has caused a significant concern everywhere throughout the world to address this issue. Nations around the globe recognized this reality with the Paris Climate Agreement of 2015. The progressions will be generally significant among the greatest producers. Twenty countries which are accountable for at least three-quarters of the GHG emissions worldwide, with the United States, India, and China paving the way, which are supposed to reduce 60 percent of CO_2_ emissions in 2030 compared to 2015.

Averting the escalation in CO_2_ concentrations is seen as imperative to reducing the risks of global warming. The economic aspect and commendable separation of CO_2_ from gas mixtures is one of the key technical and environmental challenges currently facing our society. Several capable and proven methods of CCS—such as chemical or physical adsorption [3], membrane [4], cryogenic separation [5], and hydrate-based gas separation (HBGS) [6]—have been considered to cut down the amount of CO_2_ given out to the atmosphere. At present, the post-combustion approach has been broadly used in many industries where CO_2_ is segregated by amine-based absorption techniques from flue gas. The amine-functional group’s employment is attributed to the stable chemical interaction between acidic CO_2_ and basic amino molecules. Nevertheless, its implementation leads to several adverse impacts—such as device corrosion, substantial energy usage, and solvent degradation in the presence of impurities—and is harmful to human health [7]. As a result, substitute material is much needed to overtake the constraints of this current innovation. Adsorption using solid material is attractive among the feasible CO_2_ reduction technologies owing to reduced energy prerequisites, low operating expenditure, including reduced waste production. Activated carbon (AC) is a carbon containing, immensely porous media that has an intricate structure possessed mainly of carbon atoms with greatly porous arrangement of angles, fractures, and crevices within the carbon layers. Thanks to its distinctive advantages, for instance inexpensive, reasonable for regeneration, lethargic over the moisture presence, high adsorptivity in surrounding conditions, high specific surface area and mechanical strength, adequate pore size dissemination, including low usage in energy, AC has been closely considered in many diverse processes especially in gas and liquid stages. Spent AC are categorized as hazardous solid waste and are thus forbidden to be disposed of directly at landfill sites. Since the regeneration process is more economical and environmentally sustainable than replacing a new one, it should be considered. Pressure swing adsorption (PSA), temperature swing adsorption (TSA), vacuum pressure swing adsorption (VPSA), and temperature/vacuum swing adsorption (TVSA) are some of the common regeneration technologies that industries can use to fulfil their specific objectives for air separation, gas purification, carbon capture, and VOC recovery [8]. Depending on regeneration techniques employed for adsorbent, capital investment, ease to regenerate, and flexibility to adapt to different operating situations are some of highlights need to be considered in real applications over other technologies.

Many AC materials are manufactured based on fossil-fuel means, but due to their constraint and non-sustainable qualities as primary issues therefore, origins from biomass or waste from industries have been vastly considered now days. The use of industrial and agricultural by-products for AC production—including palm kernel shell [9], coconut shell [10], paddy husk [11], banana skin [12], wood sawdust [13], etc.—has lured attention from economic and environmental aspects. The required properties of AC—i.e., large specific surface area and porosity, high carbon capacity, and exceptional sorption capability—can be altered and enhanced during thermochemical processing and activation. Although rubber plantation has shown decreased in trends, Malaysia is still the world’s main manufacturer of natural rubber (*Heavea Brasiliensis*) and produces about 46% of the world’s total rubber. According to Malaysian Natural Rubber Industry (MNRI), the production of natural rubber in 2019 is about 61,731 tonnes over 1,077.92 (‘000 hectare) but only the rubber tree milk is utilized as a primary product. Apart from this, hard shells of rubber seeds were generated simultaneously. Around 1000 kg of rubber seeds per hectare per year is expected to be generated in the rubber estate sector. The raising waste of rubber seed is creating considerable disposal as well as environment concerns. The rubber-seed shell (RSS) is recommended to be utilized as an AC material for CO_2_ removal in order to mitigate the waste dumping problem, as there is limited investigation into the management of the problems.

From an industrial point of view, size of AC plays important roles in the CO_2_ removal process when carried out in different reactor configurations such as in the fixed, fluidized bed, etc. In general, powdered activated carbon (PAC) has a particle size diameter of about 0.05 mm while granular activated carbon (GAC) has about 0.5 mm of particle size. Although fine particles, especially in the form of PAC are highly favoured due to their high surface area, tenable porosity, and excellent surface properties; the use of fine AC, however, would result in high pressure drops in a fixed-bed adsorber, therefore requiring an initial stage of pelletization process for the material. This approach may have a negative impact on the adsorption process as it can cause a substantial decrease in the sorbent specific surface area and contribute to additional resistance to intraparticle diffusion [14]. Therefore, to minimize such effects and in line with industrial operation and demand, it has decided to choose AC with particle size of 250 µm which is closer to the GAC classification throughout the investigation.

In general, AC are prepared using either chemical or physical activation methods. Typically, the raw material is combined with active agents like potassium hydroxide (KOH), zinc chloride (ZnCl_2_), and phosphoric acid (H_3_PO_4_) [12,15] when AC is chemically activated, followed by heat treatment at temperatures between 400 °C and 900 °C, where carbonization and activation being performed simultaneously thereby modifies the chemistry of surface including porosity of the produced AC. Since most of these alkali and acids are hazardous, corrosive, and expensive, while ZnCl_2_ is unfriendly to environments and create waste disposal problem, other ‘friendly’ chemicals should be considered. Malic acid with the molecular formula C_4_H_6_O_5_ is suggested as an organic compound in this study. It is known as dicarboxylic acid, produced by living things and providing the good sour taste of the fruit, and is mainly applied in food industries as a preservative. Compared with other organic acids such as citric and acetic acid, it contains low pKa readings, less impact on the environment, and is relatively inexpensive [16].

Research on the method of impregnation using this organic acid has yet to be published. Therefore, the objective of this research is to evaluate the potential of using RSS as a starting material in the manufacture of chemically activated AC material with malic acid for the removal of CO_2_. To determine the effects of operating requirements towards RSS AC pore advancement, main effecting parameters such as impregnation ratio (IR), temperature (T_act_), and activation time (t_act_) will be explored. The highest surface area samples will be used to assess the efficiency of CO_2_ adsorption at the chosen temperature and pressure. The CO_2_ adsorption isotherm is also evaluated by utilizing several models, such as Langmuir, Freundlich, and Temkin. Eventually using purified CO_2_, samples with highest adsorption potential will be assessed using the pseudo-first, pseudo-second order, and Elovich kinetic models. The goodness of these models is assessed by the coefficient of magnitude (R^2^), which means that, when closer to unity, the model predicts a closer data value to the experimental one.

## 2. Materials and Methods

### 2.1. Materials and Preparation

The precursor is acquired from a rubber estate of the Rubber Industry Small Holders Development Authority (RISDA), situated in Chemor, Perak. The raw RSS is then cleaned to extract unwanted particles using distilled water and then left in an oven overnight to dry at 120 °C. It is then mashed to 250 µm particle size and stored in airtight plastic container. The chemicals used, including DL-type malic acid (99.9% purity), were purchased from R&M Chemicals (Selangor, Malaysia) and reagents of industrial quality. All gases purchased from Linde (Prai, Malaysia) Sdn. Bhd. were high purity gases such as, CO_2_ (99.98 percent purity), helium (99.99 percent purity), N_2_ (99.99 percent purity), and compressed air.

### 2.2. Activation and Carbonization

By applying one-step chemical impregnation method, RSS precursor was mixed with malic acid based on dry weight sample at desired ratio (1:1, 1:2, and 1:3). In order to ensure a complete reaction between the precursor and the chemical reagent, the mixing and impregnation processes were allowed to sit overnight at room temperature. In the tube furnace activation unit, the impregnated substance was then carbonised at a heating temperature set at 400, 500, 600, 700, 800, and 900 °C under a nitrogen (N_2_) gas flow rate of 150 cm^3^/min and an activation time of 60 to 120 min. In this study, in order to achieve sample reduction, the one-factor-at-a-time (OFAT) technique was incorporated where an approach to the design of experiments relating to the measurement of variables is one at a time rather than all. The manufactured AC was allowed to cool at room temperature before washing it with distilled water to eliminate excess chemicals and impurities. The produced AC was finally dried at 95 °C in an oven and placed in airtight containers for further use.

### 2.3. Characterization Studies

The best operating parameter for the production of AC from RSS was analysed by several analytical instruments. The Zeiss EVO-50 Field Emission Scanning Electron Microscope (FESEM, Carl Zeiss NTS GmbH, Oberkochen, Germany), Supra 55 VP model purchased from Carl Zeiss, (Oberkochen, Germany), is used in the analysis and comparison of RSS structural morphology images at 10–100,000 times magnification before and after activation. The instrument is also designed for the study of elementary data with energy dispersive X-ray (EDX) spectroscopy.

Micromeritics Accelerated Surface Area and Porosimetry System (ASAP, Micromeritics, Norcross, GA, USA) 2020 apparatus was used to determine the total pore volume (V_T_), specific surface area (S_BET_) and pore size distribution (D) of the prepared samples using the nitrogen adsorption–desorption isotherm data. N_2_ gas of 99.9% purity as the adsorbate is administered into the system at 77 K. Approximately 300 mg samples were degassed at 150 °C for 4 h to clean the surface of impurities prior to the analysis. The S_BET_ of the prepared AC was estimated by the Brunauer–Emmett–Teller (BET) method using N_2_ adsorption isotherm data, while the Barrett–Joyner–Halenda (BJS) model was utilized to calculate the pore size distribution [17].

The infrared spectrum (absorption or emission) of the Fourier-transform infrared spectroscopy (FTIR) of Perkin Elmer, Waltham, MA, USA, is used in this research to determine structure of RSS bonds before and after carbonization. Spectra were obtained in the range of 4000 to 400 cm^−1^ at a spectral resolution of 4 cm^−1^, and 16 scans were performed per sample using Spectrum software version 5.0.1.

Thermo Gravimetric Analyzer (TGA) (Model Netzsch STA 409, Netzsch-Gerätebau GmbH, Selb, Germany) is used to analyze the pyrolysis behavior of raw RSS and AC, respectively. About 4 g of the sample was inserted into the TGA analyzer pan and N_2_ gas was permitted to flow through the furnace to maintain inert condition at 110 °C until a stable weight is reached for measuring the moisture content. The temperature was then set at 900 °C and kept for 7 min. The N_2_ is then substituted with oxygen (O_2_) gas to burn the sample. The fixed carbon content was determined through weight loss during the burning stage. The experiment was repeated with a heating rate of 15 °C/min at 30 cm^3^/min under N_2_ gas flow at a temperature range of 30–900 °C [18].

### 2.4. Studies on CO_2_ Adsorption

Using purified CO_2_, high pressure volumetric analyzer (HPVA II, Particulate Systems, Norcross, GA, USA) provided by particulate system was used in static volumetric adsorption studies. Using a balance, around 1 g of AC is first weighed, then inserted into a 5 cm^3^ cylinder of quartz sample and degassed for 8 h under vacuum at 190 °C. A filter gasket sized 60 μm was then placed atop the sample cylinder to prevent small particles from reaching the sample valve. Prior to starting the adsorption experiments, the samples were left to cool to room temperatures. The experiment was initiated by granting CO_2_ (adsorbate) gas to interact with the AC material in the system. This is achieved by allowing the valve between the loading and the sample cylinder to open for 60 min. When the sample reaches equilibrium with the adsorbate gas, the final equilibrium pressure is recorded. These data are then used to measure the amount of gas adsorbed by the sample using Microsoft Excel macros (v.22.0.8, Norcross, GA, USA) software, by taking into account the variations between the quantities of CO_2_ injected and CO_2_ remaining in the device. All measurements were made twice, and the average was used as the end result to avoid inconsistencies.

The step is performed until the preselected maximum temperature is reached at specified temperature intervals. Thus, to establish an isotherm, each of the resulting equilibrium points (volume adsorbed and equilibrium pressure) are plotted. Four isothermal models—namely Langmuir, Freundlich, Dubinin-Radushkevich (D-R), and Temkin—were chosen, and experimental data were plotted to determine the applicable representation of the AC adsorption process based on RSS. The fittings of the selected models are evaluated by comparing the values of R^2^ that are close to unity. Table 1 explains the non-linear and linear equations of these models.

By which *P_e_* represents equilibrium pressure in bar; *q_m_* and *q_e_* represent the amount of CO_2_ adsorbed at maximum and equilibrium in cm^3^/g; *k_L_* represents Langmuir constant in 1/bar; *k_F_* in cm^3^/g.bar^1/n^ and n represents Freundlich constant; *B = RT/b_T_*; *b_T_* in J/mol; and *k_T_* represents Temkin constant in cm^3^/g.bar; *λ* represents D-R constant in mol^2^/J^2^; and *ω* represents Polanyi potential in J/mol (similar to *RT* ln(1/1 + *P*)).

### 2.5. Kinetics Modeling Studies

Several most common kinetic models ranging from the pseudo-first, pseudo-second order, and Elovich model were opted to understand the mass transfer of CO_2_ adsorption on RSS AC. Using pure CO_2_ adsorption results at 25, 50, and 100 °C the appropriateness of these models reaching experimental values will be tested. The magnitude of significance regression (R^2^) closeness to unity was assessed for the conformity of the expected adsorption capability.

The linearized Lagergren model of pseudo-first order was introduced in the 19th century and represents a liquid / solid sorption method by assuming that the rate of adsorption is proportional to the number of free active sites accessible on the adsorbent surface.

The Pseudo-first order model of the linearized Lagergren was introduced at the end of the 19th century and represents a liquid/solid system for sorption by assuming that the adsorption rate is proportional to the number of free active sites on the adsorbent surface available. Equation (1) is the representation of the model [19]

(1) $$\log(q_{eq}-q_{t})=\log q_{e}-\frac{k_{1}}{2.303}$$

where the quantity of adsorption at the specified time t and equilibrium are *q_t_* and *q_eq_*, respectively, and have a unit of mg/g. The *k*_1_ serves as the Pseudo-first-order adsorption rate constant. When this model is applicable, a graph of linear plot will be obtained. Additionally, the *k*_1_ and *q_eq_* values are derived from the plots’ slope and interception.

The pseudo-second order kinetics model was proposed by Ho and McKay [20] in the mid-1980s on the basis that the adsorption rate is proportional to the square number of free sites on the surface of adsorbent and it is shown in Equation (2)

(2) $$\frac{t}{q_{t}}=\frac{1}{k_{2}q_{e}^{2}}+\frac{1}{q_{e}}t$$

where the amount of adsorption at equilibrium and at particular time *t* represented by *q_e_* and *q_t_* respectively. *k*_2_ is the overall adsorption rate constant of the pseudo-second order and its unit in g/mg.min. A linear plot will be obtained from the *t/q_t_* versus time graph, *t*, which is identical to the first order, while *q_e_* and *k*_2_ values are determined from the slope and intercepts of the plots.

The empirical adsorption model of Elovich is widely applicable to different adsorption systems and is based on the idea that sites of adsorption are heterogeneous in energy in the form of a rectangular distribution [19]. The model is stated in Equation (3) below

(3) $$q_{t}=\frac{1}{\beta }\ln\left(\alpha \;\beta \right)+\frac{1}{\beta }\ln t$$

where *q_t_* is the CO_2_ adsorbent quantity at a given time, *t*. *α* depicts the adsorption rate initially with unit in mg g^−1^ s^−1^ while *β* is the constant of the adsorption in g/mg and the energy of process activation.

## 3. Results and Discussion

### 3.1. Elemental Composition of RSS

Table 2 provides the corresponding elementary content in terms of weight percentage, before and after activation of RSS. From the EDX spectroscopy analysis, the major components of the samples detected were carbon and oxygen while hydrogen, sulphur, and nitrogen were present as minor components. Carbon content detected is 51.4% before activation while 69.4% after activation. This is in agreement with most literatures that for biomass raw material to be a prospective AC, its suitable range of carbon content, must be 40–80% [19] accordingly, it means that RSS meets AC development requirements. It is clear that, after carbonization and activation, the percentage of carbon content increased due to the release of more volatile material into gases, i.e., carbon monoxide (CO) during the development process of carbon-rich AC.

As expected, the oxygen and hydrogen content of water hydrochars decreased with processing time and temperature. Other components such as sulphur, nitrogen, and calcium have a low percentage ranging from 0.1% to 3.1% and are treated as comparably low. Zofia et al., [21] described that the presence of nitrogen in RSS surface would promote the interaction between the carbon surface and CO_2_ molecules via hydrogen-bonding, thus increasing its adsorption. A small percentage of calcium content is anticipated and can be regarded as natural in the RSS as reported by Lukman et al., [22] stated that for a 200 g serving of rubber seed can provide about 1.70 g calcium, which contributes 16% of adequate intake requirement for human bodies.

### 3.2. Surface and Porosity Analysis

The operating parameters and findings of AC samples obtained from RSS impregnated with malic acid are provided in Table 3. Highest specific surface area values, S_BET_ at 938.61 m^2^/g, average pore diameter, D at 1.368 nm and total of pore volume, V_T_ at 0.41 cm^3^/g was produced by sample A6 when operated at impregnation ratio (IR) of 1:2, T_act_ of 600 °C and t_act_ of 120 min, respectively. Samples A5 at 832.24 m^2^/g, 1.214 nm, and 0.31 cm^3^/g, and A8 at 730.61 m^2^/g, 1.330 nm, and 0.31 cm^3^/g succeeded in the process, respectively. It can be clearly seen the effect of malic acid concentration used in impregnation stage is indisputable. Samples A1 and A2 carried out at an IR of 1:1 demonstrate lower S_BET_, V_T_, D, and micropore percentage values compared to A3. Malic acid concentration does not only influence the pore volumes and the surface area but also alter the pore character and size distribution of the AC. The increase in malic acid IR to RSS from 1:1 to 1:2 in Table 3 would result in an increase in the S_BET_, V_T_, D, and micropore values. This is associated with faster reaction and intercalation between malic acid and carbon surface [12,18] resulting in increased release of CO_2_ and CO gasses leading to the formation of micropores within the mesopores. The results of this study also show that there is a limit on the maximum quantity of ions that can be allowed which would further reduce porous development. Excess activating agents is believably damaging the micropores on the carbon surface by forming an additional layer or skin covering the AC materials thus affecting the activation process. This situation is especially noticed in samples A4 and A7 where most probably activation is hampered due to high IR at 1:3, causing in significantly reduce in S_BET_, V_T_, D, and development of micropores to mesopores.

The second important parameter that affect quality of biomass-based AC produced is T_act_. Surface characterization of an AC are the crucial properties for a specific application. It has been reported by early researchers that the favorable T_act_ range is between 500–900 °C. For this reason, the experiments were performed at different temperatures from 400 to 900 °C and, according to the results from samples A1 to A2 and A5 to A11, it appears that the proposed temperature is well-founded. Sample A1, carbonized at 400 °C as starting temperature, generates the lowest S_BET_ of 265.51 m^2^/g, V_T_ of 0.12 cm^3^/g and microporal formation percentage compared with other samples. According to Wei et al. [23], the beginning T_act_ in the fundamental generation of AC material pores was recorded to be 400 °C. By raising the T_act_ from 500 to 600 °C would escalate the exclusion of volatile compounds from molecular weight and further create new pores, ensuring advanced porosity development of the AC. At these temperature ranges there seems to be S_BET_, V_T_, D, and micropore percentage enhancements where the highest is represented by sample A6 (S_BET_ of 938.61 m^2^/g, D of 1.368 nm, V_T_ of 0.41 cm^3^/g and micropores percentage of 85.24%). The reduction in pores caused by the rise in temperatures from 700 to 900 °C is attributable to the excessive heat energy supplied to the carbon material and the outcome is the destruction and knocking of some porous walls, thus deterring the creation of porosity [24]. As a result, decrease in S_BET_, V_T_, D, and micropore percentage were observed by analyzing sample A11 with other samples’ result.

In determining the quality of AC produced, the duration of AC samples allowed to be activated in a furnace also plays an important role. During the carbonization process, prolonged t_act_ can result in over-activation, where surface degradation works faster than the formation of pores. Samples A7 and A11 appear to be rapidly decreasing in percentage content of S_BET_, V_T_, D, and micropores when conducted at 180 min. It can be said that the well-established porous structure occurred at 600 °C at t_act_ of 120 min. Thus, any extension in t_act_ will result in carbon formation to fracture between its structures, following in pore disintegration [25,26]. Sample A6 is identified as the optimum sample as it has the highest S_BET_, V_T_, D, and micropore volumes while sample A11 has the lowest characterization results of all. When comparing sample A6 in this study to the one prepared by Borhan et al. [15] using similar precursor RSS but chemically activated with KOH, it was found that RSS AC with potassium hydroxide (KOH) has higher S_BET_ of 1129.60 m^2^/g compared 938.61 m^2^/g in this study. However, using malic acid as activating agent has resulted lower T_act_ at 600 °C compared to 700 °C using KOH and higher micropore volumes of 85.24% compared to 31.67%, respectively. One possible explanation for this is the low vaporization temperature of malic acid at 225 °C compared to 380 °C for KOH which makes it easy to release volatile component at low temperatures during texture formations.

**Figure 1:**
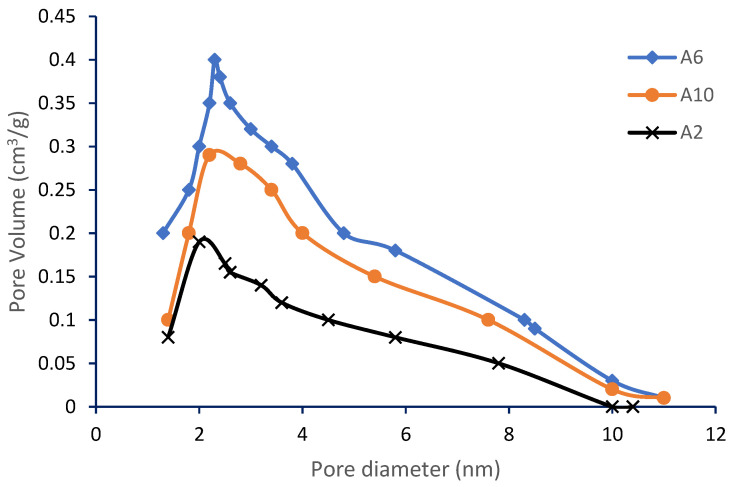
Pore size distribution of selected samples from RSS AC.

Yield can be described as the ratio of AC mass produced to the raw material mass (dry weight basis). The yield percentage of samples A3 to A9 show no significant different using malic acid when T_act_ between 500 to 800 °C. However, its value drops slightly at 900 °C due to further volatile functional groups being separated at higher temperature. In this study, the yield of RSS activated with malic acid exceeds than other biomass recorded by Kalu et al. [27] indicates it has a good prospect for AC materials. Figure 1 shows the pore size distribution of selected RSS AC namely samples A2, A6, and A10.

They are ranked in the order of declining pore volume from top to bottom. As shown in the figure, they are all categorized under micropore volume while the highest pore volume belongs to sample A6. The T_act_ can be seen has had a major influence on the pore structure of the prepared AC. The pore structure consisted primarily of micropores at low and moderate temperatures (at 500 °C for A2 and 600 °C for A6). The development of mesopore increased, with the increase in T_act_ and the overall pore volume of AC was increased by this. The reaction rate between the activating chemical and carbon has been shown to increase geometrically as the temperature increases. Micropores were expanded at high temperatures and the walls between pores collapsed and thus mesopores formed. Pore size can be characterized as macro if the size is >50 nm, meso if the size is between 2–50 nm and micropore if the size is <2 nm, as described by the International Union of Pure and Applied Chemistry (IUPAC) [26]. All samples except raw, A1, A10, and A11 exhibit a pore diameter of less than 2 nm, thus clearly categorizing the pores as belonging to the classification of micropores.

### 3.3. Surface Morphology

**Figure 2:**
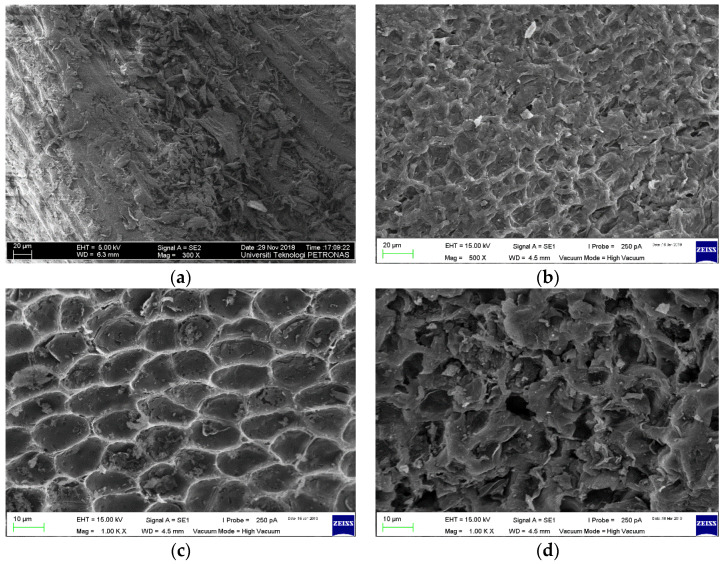
FESEM micrographs of selected samples, (**a**) Raw RSS, (**b**) sample A1, (**c**) sample A6, and (**d**) sample A9.

The morphological structure of the raw RSS and the three selected ACs prepared is represented in Figure 2a–d with magnifications up to 100,000 times at different parameters using FESEM. The FESEM micrographs exhibit a significant difference in the raw biomass sample surface morphology without activation and after activation of the optimized samples. The structural morphology of raw RSS shown in Figure 2a reveals very minimum existence of pores on the relatively flat smooth surfaces, which is one of the crucial features for preparing AC.

The emergence and improved distribution of new pores on RSS AC surfaces is credited to the elimination of volatile matters following carbonization process. By comparison of Figure 2a–c, the variation in pore structure before and after activation can evidently be displayed. Following the activation of malic acid, more porous structures can be seen due to the dehydration effect of malic acid and the oxidation of organic compounds during sample A1 carbonization phase. Further increased in T_act_ and t_act_ resulted in sample A6 being produced with higher S_BET_, V_T_, and D than sample A1. Figure 2c showed an irregular and the most well-evolved porous structure than the other three. Breakdown of porous wall with cracks and crevices due to immoderate heat exposure reduced the surface area and adsorption capacity at highest temperature in Figure 2d with activation of sample A9. This observation that the growth in the number of meso- and macro-pores when biomass AC is subjected to high temperature is supported by Couto et al. [28].

### 3.4. FTIR Analysis

**Figure 3:**
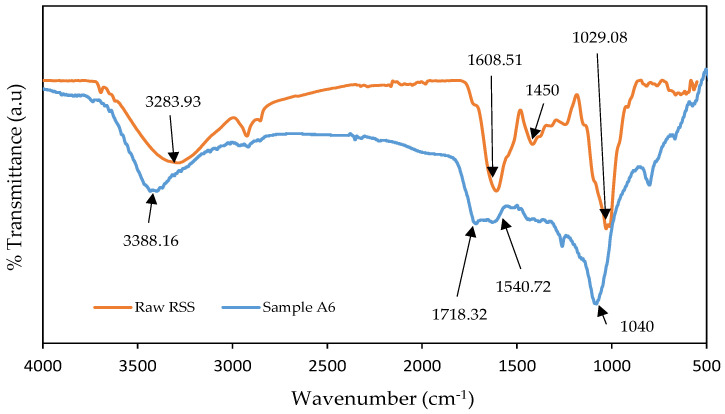
FTIR spectra of raw RSS and Sample A6.

Figure 3 displays the combined FTIR spectra analysis for raw RSS precursor and sample A6. It can be noted from the FTIR analysis of the samples that almost all of them shared similar peaks and values to support the fact that they are the same sources. There is a strong wide adsorption peak showed at 3283.93 cm^−1^ associated with the functional group of O-H or N-H stretching and this designated the presence of the hydroxyl group in the raw RSS. The band detected at 1608.51 cm^−1^ is associated with C=C stretching in aromatic ring and summit around 1450 cm^−1^ signifies the possibility of the existence of pyrones and aromatic groups. The raw RSS also shows important peak of adsorption at 1029.08 cm^−1^, attributed to the functional group of C-O stretching. The weak peaks of around 882 cm^−1^ and 768 cm^−1^ suggest out-of-plane bending of the C-H group in the aromatic ring. Therefore, the trend of the FTIR spectrum analysis for the raw RSS disclosed a complex surface due to the presence of several peaks.

Figure 3 also displays the sample A6 FTIR spectra after carbonized with malic acid at IR of 1:2, 600 °C for 120 min. Although the T_act_ is increased to 600 °C, most of its organic group were still present as in raw RSS but with increasing transmittance intensity in some peak areas. The FTIR peak intensity is proportional to the number of functional groups. It is normal that increase or decrease occur in some of the FTIR peaks after treatment, particularly after chemical treatment. This shows that some restructuring of the surface oxides occurred at high temperature upon subjection to activation. This is in line with report by Mohtashami et al. [29] where the carbonized and chemically activated samples using H_3_PO_4_ exhibit higher peaks and transmittance intensity than that found in the raw sugarcane bagasse. It is observed that several new functional groups are also detected, between 1620–1850 cm^−1^ wavelength with actual peak at 1718.32 cm^−1^, strong C=O stretching indicate presence of carboxylic acid, and medium O-H bending at 1540.72 cm^−1^ attributable to carboxylic acid as well, possibly from usage of malic acid in the experiment. Since some of the functional groups sensitive to temperature are released, the use of malic acid activation could somehow prevent the functional groups in the RSS from diminishing entirely.

All samples have a band associated with O-H stretching vibration of hydroxyl functional group. According to study made by Jiangtao et al. [30], the presence of oxygen in functional group can assists with the hydrogen bonding interaction between the surface and CO_2_ molecules. This acidic condition caused by an oxygen containing group will eventually enhance the CO_2_ uptake by the carbon surface.

### 3.5. TGA Analysis

**Figure 4:**
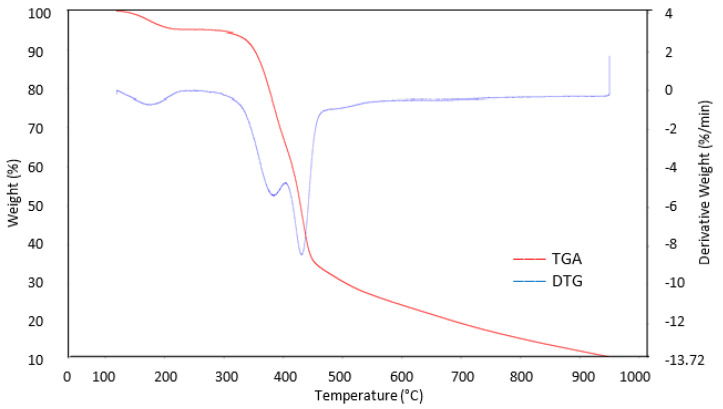
TGA and DTG profiles of raw RSS.

**Figure 5:**
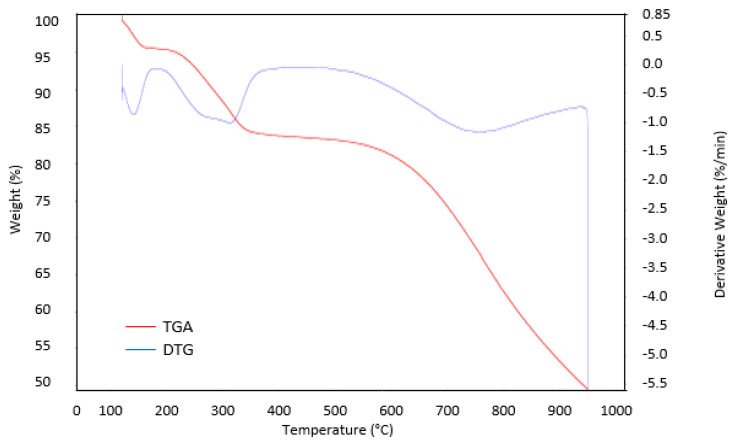
TGA and DTG profiles of sample A6.

To study their decomposition properties, a thermal analysis of the raw RSS and optimum AC sample A6 were conducted and their TGA and derivative thermogravimetry (DTG) results are shown in Figure 4 and Figure 5, respectively. Three stages of weight loss appeared in the raw RSS.

At first stage about 10.3% weight loss in raw RSS in a temperature range of 120–230 °C, which might have influenced from the unleash of moisture content and surface bounded water. Throughout the second stage, at temperature range of 325–430 °C, steep weight loss and upmost at 53.5% was recorded. This huge weight loss may be correlated to the breakdown of hemicellulose and elimination of volatile matters (i.e., CO, H_2_O, H_2_, and CO_2_) and low molecular weight constituents (i.e., acetic acid, wood tar, and methanol). This is also an indication that pyrolysis has occurred at this temperature scale. Normally solid dark leftovers were obtained but with incomplete carbonization phase. The third stage involved at temperature of 500 °C which may be associated to the decomposition of cellulose and at this point, the material becomes rich in carbon content with rudimentary pores developed within the matrix. The process further progresses beyond 900 °C due to the disintegration of lignin with about 18% weight loss result in mixture of ash and carbon. The TGA sample A6 revealed final weight loss of less than 49.8% compared than the raw RSS. It can be deduced that raw RSS was the least thermally stable compared to the one activated with malic acid. Sample A6 in Figure 4 has three decomposition regions as raw RSS, but with less steep weight loss. Although decomposition of cellulose and hemicellulose including the volatile components occurred in sample A6 during the second stage, the presence of carboxyl (COOH) and carbonyl (C=O) group of malic acid residue attached to the carbon surface probably helps to prolong the decomposition of material from increase in temperature effect until it reached 630 °C. This can be shown from the almost constant line and slow rate of weight decomposition of sample A6 at temperature range of 330–610 °C with less than 10% weight loss. Therefore, the formation of pores with the highest S_BET_, V_T_, D, and micropores volume were recorded for sample A6.

### 3.6. CO_2_ Adsorption Performance and Isotherm Studies

**Figure 6:**
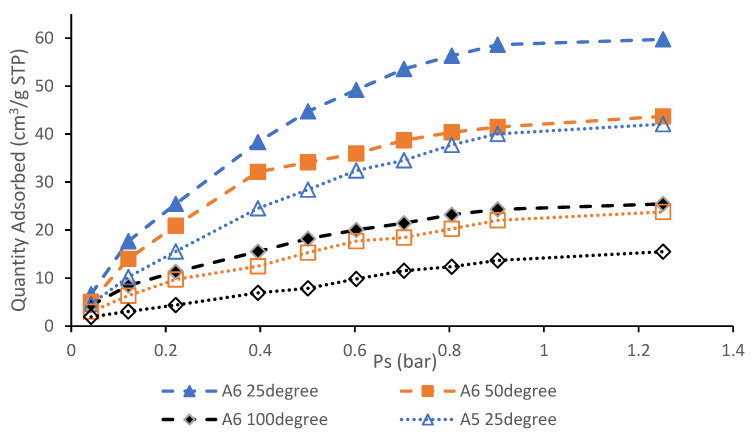
CO_2_ adsorption profiles of samples A5 and A6 at 25, 50, and 100 °C.

Figure 6 shows CO_2_ adsorption profiles for the A6 and A5 samples at different temperatures. The quantity of CO_2_ adsorbed is plotted against pressure at three different temperature values. It was reported by Garcia et al. [31] that in post-combustion CO_2_ capture, in order to facilitate adsorption and to maintain material reliability, the temperature has to be low (<100 °C). Therefore, adsorption experiments at 25, 50, and 100 °C were conducted to examine the CO_2_ adsorption behavior at any of these raised temperatures.

It can be shown that in the isotherm adsorption, all lines display common patterns which is Type I according to IUPAC classification [25] where it implies the presence of micropores within the surface of AC. Once fully filled with CO_2_ adsorbents, very little or no exterior surface is left for additional adsorption. The concave curve of the pressure axis shows that its limiting value is reached by the amount of CO_2_ adsorbed. A steep uptake at the beginning of the cycle at low-pressure is caused by the increased adsorbent–adsorptive correlations in narrow micropores (molecular-dimensional micropores), leading to the relatively low-pressure stuffing. At 25 °C and 1.25 bar for sample A6, the highest CO_2_ adsorption potential was 59.73 cm^3^/g (107.99 mg/g), which was greater than 42.06 cm^3^/g in sample A5 at 25 °C and 1.24 bar, respectively. It undoubtedly demonstrates that the capability of CO_2_ adsorption decreases with respect to temperature due to the decline in binding strength between the adsorbate of CO_2_ and AC. Because of the physisorption mechanism, the adsorption of CO_2_ into AC is exothermic and, according to Estaves et al. [32], the decrease in the adsorption capacity of CO_2_ at high temperatures shows that the process of sorption is exothermic as it contains a weak van der Waals force that is likely to be destroyed at high temperatures and thus reduces the ability to adsorb. High adsorption temperature provides high molecular diffusion speed and higher surface adsorption energy due to instability of CO_2_ adsorbates on the carbon surface, CO_2_ molecules are released (desorption) from the surface. In summary, decline in CO_2_ adsorption capacity concerning to the temperature indicate exothermic process and is administered by physisorption.

Sample A6 which has higher adsorption capacity of CO_2_ than A5 is shown in Figure 6. These outcomes are in line with the analysis performed using Micrometrices ASAP 2020 for surface and porosity analysis of AC samples where higher S_BET_, V_T_, D, and micropore volume does lead to higher adsorption capacity. Another important parameter which is operating pressure is also examined where increase in pressure leads to higher CO_2_ adsorption. This occurrence is likely to happen due to the high pressure that likely forces CO_2_ molecules onto adsorption sites inside the pore [33]. Highest CO_2_ adsorption pressure recorded by samples A6 and A5 are at 1.25 and 1.24 bar, respectively. Since sample A6 has the largest S_BET_ and V_T_ this means more surface sites and volume are available to adsorption processes.

Table 4 displays the measured parameters and their respective R^2^ values of sample A6 when four model of Langmuir, Freundlich, Dubinin-Radushkevich, and Temkin are adjusted to the experimental results. The Langmuir *k_L_* and the Freundlich *k_F_* constant are associated with the adsorption affinity energy, where its value decreases as temperature increases, which suggests an existence of physisorption according to Romero et al. [19]. Increasing trends are shown from increasing of temperature from 25 to 100 °C for both *k_L_* and *k_F_*, respectively. The value of *q_m_* is expected to decrease with the increase in adsorption temperature, which explains the exothermic nature of adsorption of CO_2_. The Langmuir model considers that the molecules are adsorbed to a specified number of active locations forming a single monolayer with the same energy possessed by the adsorption site. Freundlich adsorption model is based on different adsorption energies which result in an exponential decrease in energy as surface coverage originates from adsorption at different sites and multilayer formation [33]. A variation in the linearity of the adsorption potential is expressed by the Freundlich constant heterogeneity factor, n, and is used to determine the adsorption favorability form.

The value of *n* > 1 indicates physical adsorption while *n* < 1 corresponds to chemical adsorption. The Dubinin-Radushkevich adsorption model was established to include for the effect of the porous structure of the adsorbents while for Temkin isotherm model, it is based on the enthalpy of adsorption of all the molecules on the surface decreases linearly with coverage due to adsorbent–adsorbate interactions [34]. In addition, these two isotherms will provide a valuable data related to the energy specification, in terms of mean free energy of adsorption, E and heat of adsorption, bT. The determined E values which are within the range of 3–4 kJ/mol indicates that the CO_2_ undergo physical adsorption, as the magnitude of E is below 8 kJ/mol, while value between 8 and 16 kJ/mol indicates chemical adsorption [33,34,35]. It was noticed that the value of R^2^ was at a range between 0.9910 to 0.9951 for Freundlich isotherm, 0.9744 to 0.9873 for Langmuir isotherm, 0.9432 to 0.9611 for Dubinin-Radushkevich, while the Temkin isotherm was between 0.9305 to 0.9589. Based on R^2^ value approaching unity, the Freundlich model gives the best fit towards the experimental data over the entire temperature range. This indicates that the surface of AC is heterogeneous and allows multi-layer adsorbate CO_2_ molecules to shape a continuous layer onto the surface of AC.

### 3.7. Kinetic Analysis of the CO_2_ Adsorption Process

**Figure 7:**
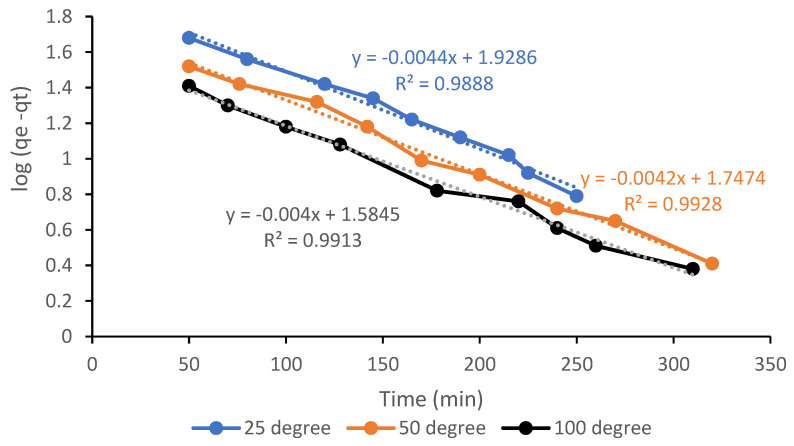
Pseudo-first order CO_2_ adsorption kinetics on sample A6 at 25, 50, and 100 °C.

The process of adsorption relies primarily on the potential of the synthesized AC for accumulating CO_2_ molecules through physicochemical passage. For kinetic studies, an assessment of the mechanism such as mass transfer is therefore vitally important [19,20]. Apparently, the adsorption mechanism has to be confirmed by testing the correct kinetic models. Using previously optimised sample A6, the CO_2_ adsorption kinetics on RSS-derived AC were examined and the results were tested using three existing models, namely the pseudo-first order, pseudo-second order, and Elovich models. In principle, any of these three models may be used to characterise the CO_2_ adsorption on the evaluated AC. For this adsorption process, however, not all models may be equally appropriate. The experimental values are presented by the points on the plot while the dashed lines represent the quantity predicted by matching the experimental data to the models of kinetic. A log plot (*q_e_−q_t_*) against *t* at 25, 50, and 100 °C for sample A6 resulted in linear graphs with negative slopes for all pseudo-first order temperatures is shown in Figure 7. The intercepts and gradients of the plots were used to calculate the constant of the first order rate, *k*_1_, and the capacity of equilibrium adsorption, *q_e_*. The *k*_1_ and *q_e_* values decreased with temperature rise from 0.0821 to 0.0068 min^−1^ and from 138.7160 to 74.929 mg/g, respectively. It is also noticed that during the adsorbate–adsorbent interactions, the amount of CO_2_ adsorbed on the adsorbents decreases with an increase in the adsorption temperature due to a decrease in the adsorbent density. According to Estaves et al. [32], the amount of CO_2_ adsorbed on the adsorbents is rapid at the beginning and steadily reduced as the process proceeds until the equilibrium state is reached. It was found that CO_2_ molecules come into direct interaction with the adsorbent AC at the initial level, resulting in a stronger binding force. CO_2_ molecules can no longer be adsorbed once the adsorbate inhibits all pores. This is in agreement with the assumption of the model where the rate of adsorption is proportional to the number of free active sites on the adsorbent surface available. From a summary of Table 5, the pseudo-first order model was found to match with the CO_2_ adsorption profile with regression coefficient values for the three temperature values at 0.9888, 0.99288, and 0.9913, respectively. Romero et al. [19] published similar findings when using pseudo-first, pseudo-second, and modified pseudo-first order models to analyze the quantity of CO_2_ adsorption at 0 °C on resorcinol-formaldehyde aerogels, where the predicted pseudo-first result was much nearer and more consistent with the experimental values.

**Figure 8:**
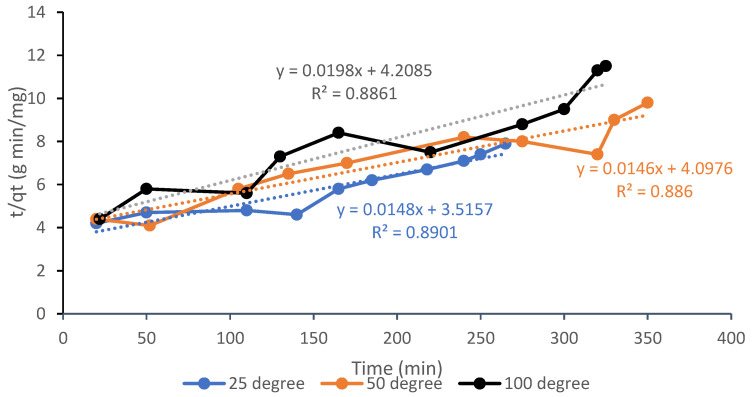
Pseudo-second order kinetics adsorption for CO_2_ on sample A6 at 25, 50, and 100 °C.

For the pseudo-second order kinetics model, Figure 8 shows a graph of *t/q_t_* versus *t*. If the model is applicable to the adsorption process, it will produce a straight line with 1/*h* and 1/*q_e_* as y-interception and slope, respectively. The R^2^ values of the three temperatures between 0.8710 to 0.8922 is considered low compared to the pseudo-first order within range of 0.9913 to 0.9928, therefore is not suitable to be utilized in the adsorption kinetics process although researchers have widely used it to model the kinetics of experimental CO_2_ data [33,34]. The *k*_2_ constant values showed increasing trend from 1.09 × 10^−4^ at 25 °C to 1.74 × 10^−3^ at 100 °C with the rise of adsorption temperature indicating exothermic behaviour that the adsorption is preferred at lower temperatures. Also, the magnitude of *h* which served as rate of adsorption was noted to decrease with regard to temperature, indicating the rapid rate of CO_2_ adsorption during the initial period of time, and then it slowed as it progressed. According to Singh and Kumar [35] on adsorption kinetic of CO_2_ on AC Norit RB3 and zeolite 5A at various temperatures and pressures, the weak van der Waals forces can be overcome by high-energy CO_2_ molecules at high temperatures and eventually break free from the AC surface and return to the bulk gas phase.

**Figure 9:**
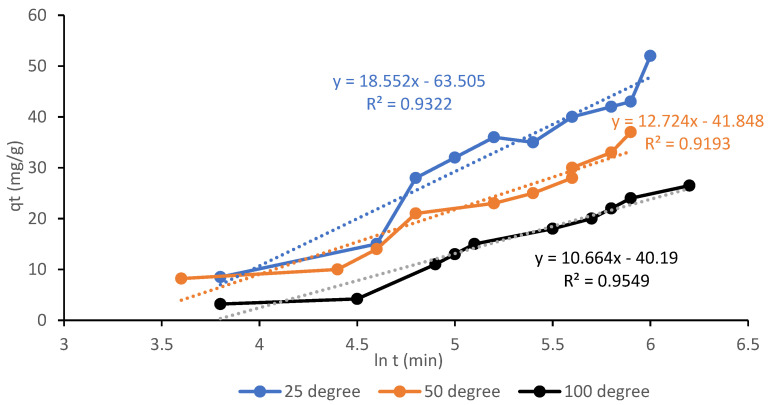
Elovich kinetics adsorption for CO_2_ on sample A6 at 25, 50, and 100 °C.

Using the Elovich model shown in Figure 9 with R^2^ values ranging from 0.9193 to 0.9549, the second-best match was achieved with the experimental results. The *α* values indicate the initial speed of adsorption where its value decreased with temperature, with values of 0.6282, 0.4028, and 0.0932 mg/g.min from 25, 50, and 100 °C, respectively suggesting that the initial adsorption rate under these conditions is related to the CO_2_ adsorption capacity. The trend is similar to the initial adsorption rate, *h* of pseudo-second order model. For sample A6, the value of constant *β* has the smallest value (0.0390 g/mg) thus indicating that the AC has the highest CO_2_ adsorption capacity since it had the highest volume of micropores. The reverse adsorption process (desorption) is also illustrated by this model, as the value of *β* describe the desorption rate. It can be identified from Table 5 that the value of *β* increases from 0.0390 g/mg at 25 °C to 0.9243 g/mg at 100 °C, suggesting regulation of desorption. As shown from the table, the kinetic model of Elovich poorly fitted into the experimental data compared to the pseudo-first order due to the regression coefficient values but performed much better than the pseudo-second order model.

To sum up, the pseudo-second order and Elovich model are less suitable when placing the results of CO_2_ adsorption kinetics because of their low R^2^ values. Notable differences are found when applying these two models to measure the adsorption capacity. The pseudo-first order model fits the kinetic adsorption data with R^2^ values between 0.9888 and 0.9928 over the investigated temperature range, suggesting that the adsorption inclines towards physisorption while that pseudo-second order achieved the lowest R^2^ value which correlates to the absence or little chemisorption activity between the CO_2_ and the AC surface. The suitability of the three kinetic models are as follows: pseudo-first order > Elovich > pseudo-second order.

In any adsorption process, the activation energy capacity, *E_a_* is essential because it offers valuable information of its mechanism. According to Singh and Kumar [35] and Shahkarami et al. [36], since physical adsorption consists of a weak adsorbate–adsorbent bond, the *E_a_* value is typically about 5 to 40 kJ mol, whereas the chemisorption process is between 40 and 800 kJ/mol, respectively. Due to weak van der Waals forces between adsorbate–adsorbent interactions, the value of *E_a_* is generally low in the physisorption process. For determining the *E_a_* of the adsorption process, the Arrhenius equation as shown in Equation (4) is used.

(4) $$\ln k=\frac{-E_{a}}{RT}+\ln k_{o}$$

where *k* is the rate constant of the kinetic model of Pseudo-second order, unit in g/mg∙min, *E_a_* is the activation energy of adsorption, unit in J/mol, *R* is the gas constant, unit in 8.314 J/mol∙K, *T* is the temperature of adsorption, unit in Kelvin, and *k_o_* is the factor of temperature independent, unit in g/mg∙min. In the present work, it is found that the *E_a_* of sample A6 is 19.41, 16.38, and 9.37 kJ/mol at 25, 50, and 100 °C, respectively and a similar trend was recorded by Singh and Kumar [35]. The *E_a_* values for sample A6 AC are in the range of that of physisorption processes, thus implies that the CO_2_ adsorption is a diffusion-controlled, instead of chemically controlled.

### 3.8. Comparison Study with Other Adsorbent Materials

A comparison of the adsorption capacity of CO_2_ onto different types of AC ranging from agricultural/domestic waste to Norit^®^ SX2 and Zeolite 13X commercial activated carbon (CAC) is shown in Table 6. It can be seen from this comparative study that sample A6 which is derived from RSS AC has a significantly greater CO_2_ adsorption capacity potential than coconut shell char, sawdust, banana peel, sewage sludge, rice husk, palm kernel shell, coconut shell, RSS activated with KOH, Rht-MOF-7, CAC of Zeolite 13X, and Norit^®^ SX2. The highest adsorbent is SiFSIX-3-Zn AC a type of metal organic material (MOM) which can be considered latest of its kind and is followed by bimodal silica AC with CO_2_ adsorption capacity of 124.52 and 123.23 mg/g respectively. Meanwhile in this work RSS AC is ranked third with a 107.99 mg/g adsorption intake. In addition, the amount of CO_2_ adsorption capacity with malic acid was also found to be higher than that activated with KOH [15]. This may be due to the presence of functional group of hydroxyls that assists in the hydrogen bonding interaction between the CO_2_ molecules and their surface, thereby improving CO_2_ uptake [30]. Synthesized AC from waste material has an adsorption rate close to or noticeably higher than the Norit^®^ SX2 (Brenntag, Kędzierzyn-Koźle, Poland) and Zeolite 13X CAC (Jalon, Henan, China), where the latter is considered as benchmark in production of biomass-based AC. Although MOM of SIFSIX-3-Zn has the highest adsorption CO_2_ capacity; however, its starting material which is originated from non-biomass and non-renewable precursor makes it less sustainable than the other adsorbent materials. Structural instability of materials, high cost of linkers, and instability with the presence of water vapor are some of the drawbacks that this material has. Utilization of waste biomass as starting materials for AC production also ensures an environmentally sustainable and tenable passage for CO_2_ sorbent material growth.

## 4. Conclusions

This study has demonstrated the ability to convert RSS into AC from low-cost and abundant agricultural waste using malic acid as an activating agent for the removal of CO_2_ by adsorption process. Activation conditions have a significant influence on porous properties where the resulting AC of sample A6 produced a high S_BET_, V_T_, D, and micropore volume when impregnated at 1:2 with malic acid, activated at temperature of 600 °C for 120 min. Characterization analysis revealed the material belongs to the micropores classification following Type I adsorption isotherm while Freundlich model is the best fit based on the isotherm model analysis. TGA analysis has shown malic acid can reduce the thermal degradation of the AC and new functional group of hydroxyls was detected when performing FTIR analysis of the sample which eventually enhance the CO_2_ uptake by the surface of the carbon. The extracts of CO_2_ adsorption kinetics experimental results are used for mathematical modeling using three separate standard models, such as pseudo-first, pseudo-second order, and Elovich models, using statistical volumetric technique. From the best-fitted kinetics model, the adsorbent–adsorbate interactions and kinetic behavior of CO_2_ adsorption are deduced. Based on the correlation coefficient, the pseudo-first order kinetics model was found to fit very well with the experimental CO_2_ adsorption kinetics results. By fitting the Arrhenius equation to the adsorption kinetics results, activation energies at distinct temperatures are determined. It is seen from the analysis that low temperature adsorption of CO_2_ is physisorption between the adsorbate–adsorbent interaction due to van der Waals’ weak forces. In terms of effectiveness, RSS AC with malic acid is capable in lowering CO_2_ and its performance can be considered on par or superior with other already established biomass-based AC, MOF, and CAC, thus making it an interesting choice to be considered in future CO_2_ mitigation plan.

## Figures and Tables

**Table 1:** Isotherm model equations used.

Isotherm	Non-Linear Equation	Linear Equation
Langmuir	$q_{e}=\frac{q_{m}k_{L}P_{e}}{1+k_{L}P_{E}}$	$\frac{P_{e}}{q_{e}}=\frac{1}{q_{m}}P_{e}+\frac{1}{k_{L}q_{m}}$
Freundlich	$q_{e}=k_{F}P^{\frac{1}{n}}$	$\log q_{e}=\frac{1}{n}logP_{e}+\log k_{F}$
Dubinin-Radushkevich	$q_{e}=q_{m}e^{-\lambda \omega 2}$	$ln\;q_{e}=\ln q_{m}-\lambda \omega^{2}$
Temkin	$q_{e}=B\;(\ln k_{T}P_{e})$	$q_{e}=B\ln P_{e}+B\ln k_{T}$

**Table 2:** Elemental compositions comparison.

Elements	RSS before Carbonization	Sample A6 after Carbonization
	Weight (%)	Weight (%)
Carbon	51.4	69.4
Oxygen	38.5	22.1
Hydrogen	5.3	2.1
Sulphur	0.1	0.1
Nitrogen	1.6	3.4
Calcium	3.1	2.9
Total	100	100

**Table 3:** Surface characterization results.

Sample	IR	Act. Temp, T_act_ (°C)	Act. Time, t_act_ (min)	Specific Surface Area, S_BET_ (m^2^/g)	Total Pore Volume, V_T_ (cm^3^/g)	Average Pore Diameter, D (nm)	Percentage Micro Pores (%)	Yield (wt%)
Raw	-	-	-	3.05	0.00	4.554	19.82	-
A1	1:1	400	60	265.51	0.12	3.810	68.21	44.07
A2	1:1	500	60	471.14	0.19	1.084	79.04	43.85
A3	1:2	500	120	684.19	0.26	1.173	83.10	42.04
A4	1:3	500	120	502.50	0.24	1.155	81.32	42.28
A5	1:1	600	120	832.24	0.31	1.214	83.73	41.86
A6	1:2	600	120	938.61	0.41	1.368	85.24	42.72
A7	1:3	600	180	622.44	0.37	1.231	84.02	41.22
A8	1:2	700	120	730.61	0.31	1.330	84.07	40.62
A9	1:2	800	120	682.13	0.31	1.372	83.24	40.26
A10	1:2	900	120	510.20	0.30	2.214	82.92	38.66
A11	1:2	900	180	302.35	0.21	3.025	72.70	38.02

**Table 4:** Parameters of the isotherms of Langmuir, Freundlich, Dubinin-Radushkevich, and Temkin for CO_2_ adsorption of sample A6 at three different temperatures.

Type	25 °C	50 °C	100 °C	Type	25 °C	50 °C	100 °C
**Langmuir**				**Freundlich**			
*q_m_*	76.7234	59.0621	41.4431	*k_F_*	43.7640	30.8725	12.5502
*k_L_*	2.4326	1.32280	0.6844	*n*	2.7697	1.4205	1.2009
R^2^	0.9873	0.9744	0.9821	R^2^	0.9952	0.9951	0.9910
**Dubinin Radushkevich**				**Temkin**			
*q_m_*	39.7542	25.8004	12.6642	*B*	12.0744	9.2582	4.7621
*λ*	3.6771E-8	3.8261E-8	4.8011E-8	*k_T_*	28.903	19.005	13.1774
*E*	4.0241	3.4902	3.0182	*b_T_*	238.7742	389.0624	714.5542
R^2^	0.9432	0.9576	0.9611	R^2^	0.9589	0.9476	0.9305

**Table 5:** Kinetic adsorption parameters of CO_2_ for sample A6 at 25, 50, and 100 °C.

Type	25 °C	50 °C	100 °C	Type	25 °C	50 °C	100 °C
**Pseudo-First Order**				**Pseudo-Second Order**			
*q_e_* (mg/g)	138.716	101.447	74.929	*k*_2_ (g/mg min)	1.09 × 10^−4^	1.45 × 10^−4^	1.74 × 10^−3^
*k*_1_ (1/min)	0.0882	0.0294	0.0068	*h* (mg/g min)	2.097	1.276	0.9761
R^2^	0.9888	0.9928	0.9913	R^2^	0.8901	0.8860	0.8861
**Elovich**							
*β* (g/mg)	0.0390	0.0638	0.9243				
*α* (mg/g min)	0.6282	0.4028	0.0932				
R^2^	0.9322	0.9193	0.9549				

**Table 6:** Comparison of CO_2_ adsorption capacities with other waste materials.

Adsorbent Types	Activation Method	CO_2_ Adsorption Capacity (mg/g)	References
Coconut shell	Chemical (NaOH)	27.10	[10]
Banana peel	Chemical (KOH)	48.40	[12]
Rice husk	Chemical (ZnCl_2_)	57.13	[11]
Palm kernel shell	Chemical (ZnCl_2_)	62.05	[9]
Coconut shell char	Chemical (MEA)	35.57	[37]
Sewage sludge	Physical (microwave)	53.30	[38]
Sawdust biochar	Chemical (MEA)	44.80	[14]
Bimodal silica	Physical (hydrothermal)	123.23	[39]
Norit^®^ SX2 CAC	Physical (steam)	82.74	[40]
Rht-MOF-7	Chemical (MOF)	105.6	[41]
Zeolite 13X CAC	Physical (hydrothermal)	91.23	[35]
SIFSIX-3-Zn	Chemical (MOM)	124.52	[42]
RSS AC	Chemical (KOH)	54.41	[15]
RSS AC	Chemical (malic acid)	107.99	This work
